# Simultaneous Homozygous Mutations in *SLC12A3* and *CLCNKB* in an Inbred Chinese Pedigree

**DOI:** 10.3390/genes12030369

**Published:** 2021-03-05

**Authors:** Lijun Mou, Fengfen Wu

**Affiliations:** 1 Department of Nephrology, The Second Affiliated Hospital, School of Medicine, Zhejiang University, Hangzhou 310009, China; moulj511@zju.edu.cn; 2 Department of Nephrology, The Second Affiliated Hospital, School of Medicine, Zhejiang University, Long-quan Branch, Longquan 323716, China

**Keywords:** *SLC12A3*, *CLCNKB*, Gitelman syndrome, Bartter syndrome, calcium pyrophosphate deposition disease, gout

## Abstract

Gitelman syndrome (GS) and Bartter syndrome (BS) type III are both rare, recessively inherited salt-losing tubulopathies caused by *SLC12A3* and *CLCNKB* mutations, respectively. We described a 48-year-old male patient with fatigue, carpopedal spasm, arthralgia, hypokalemic alkalosis, mild renal dysfunction, hypomagnesemia, hypocalciuria, hyperuricemia, normotension, hyperreninemia and chondrocalcinosis in knees and Achilles tendons. His parents are first cousin. Genetic analysis revealed simultaneous homozygous mutations in *SLC12A3* gene with c.248G>A, p.Arg83Gln and *CLCNKB* gene with c.1171T>C, p.Trp391Arg. The second younger brother of the proband harbored the same simultaneous mutations in *SLC12A3* and *CLCNKB* and exhibited similar clinical features except normomagnesemia and bilateral kidney stones. The first younger brother of the proband harbored the same homozygous mutations in *CLCNKB* and exhibited clinical features of hypokalemia, normomagnesemia, hypercalciuria and hyperuricemia. Potassium chloride, spironolactone and potassium magnesium aspartate were prescribed to the proband to correct electrolytic disturbances. Benzbromarone and febuxostat were prescribed to correct hyperuricemia. The dose of potassium magnesium aspartate was subsequently increased to alleviate arthralgia resulting from calcium pyrophosphate deposition disease (CPPD). To the best of our knowledge, we are the first to report an exceptionally rare case in an inbred Chinese pedigree with simultaneous homozygous mutations in *SLC12A3* and *CLCNKB*. GS and BS type III have significant intrafamilial phenotype heterogeneity. When arthralgia is developed in patients with GS and BS, gout and CPPD should both be considered.

## 1. Introduction

Gitelman syndrome (GS) and Bartter syndrome (BS) type III are both rare, recessively inherited salt-losing tubulopathies (SLTs), characterized by hypokalemic metabolic alkalosis with low or normal blood pressure, despite secondary hyperreninemia and aldosteronism [1]. GS is caused by loss-of-function mutations in the *SLC12A3* gene, encoding the thiazide-sensitive sodium-chloride cotransporter (NCC) apically expressed in epithelial cells of the distal convoluted tubule (DCT). BS type III is caused by loss-of-function mutations in the *CLCNKB* gene encoding the chloride channel ClC-Kb [1,2]. GS is a milder disease compared with BS, frequently associated with hypomagnesemia and hypocalciuria [3]. Considerable phenotypic variability of BS type III has been described, including hypomagnesemia and hypocalciuria, that is, Gitelman-like (GLS) phenotypes [4]. However, clinical and biological features alone are not sufficient to differentiate GS from BS type III [3]. In some patients with GS rather than BS, calcium pyrophosphate deposition disease (CPPD) develops, probably due to long-term and profound hypomagnesemia [5]. Arthritis resulting from CPPD could be easily misdiagnosed as gout [6]. Unlike patients with GS, hyperuricemia and acute gouty arthritis are commonly seen in patients with BS rather than GS [7] and, therefore, in SLT patients with coexisting hypomagnesemia and hyperuricemia-induced arthralgia it is very difficult to identify the exact causes of arthralgia. We describe an inbred family with coexisting hypomagnesemia and hyperuricemia caused by simultaneous homozygous mutations in *SLC12A3* and *CLCNKB* gene. To the best of our knowledge, this is the first report on simultaneous homozygous mutations in *SLC12A3* and *CLCNKB* gene.

## 2. Materials and Methods

### 2.1. Case Description

On May 28th, 2017, a 48-year-old male patient (II-2) was admitted to our hospital due to fatigue for 45 years and intermittent carpopedal spasm for 30 years. From the age of three years old he suffered from paroxysmal fatigue, salting craving, polydipsia and polyuria. Laboratory exams revealed hypokalemia, and he had been taking oral potassium chloride irregularly. No palpitation, constipation or physical and mental retardation was reported. Episodic carpopedal spasm developed at the age of 18 years. Intense pain, redness, warmth and swelling in the proximal interphalangeal (PIP) joint of the right index finger developed at the age of 36 years. Bilateral knees and heels were also involved later. The arthralgia was initially diagnosed as gout and was partially resolved following acetaminophen and benzbromarone administration. However, the arthralgia flared frequently. On physical examination, blood pressure was 128/75 mmHg. There was no joint deformity or tophus in any joint. Serum biochemistry revealed hypokalemia, hypomagnesemia, hyperuricemia and mildly renal dysfunction. Arterial blood analysis showed decompensated metabolic alkalosis. Urinary electrolytes analysis demonstrated renal potassium, magnesium wasting and hypocalciuria. Renin activity was elevated (Table 1). His parents are first cousins. II-2 has three younger brothers, none of whom has married. He had a son. The blood pressures of his family members were normal. I-1 (father) has mild hypokalemia with renal potassium wasting, gout, mild renal dysfunction and left kidney stone. I-2 (mother) has type 2 diabetes without any electrolytic disorders. II:-3 (the first younger brother) and -II-4 (the second younger brother) both have hypokalemia with renal potassium wasting and normomagnesemia. Besides, II-4 has mildly renal dysfunction, hypocalciuria, bilateral kidney stones, right kidney cyst and type 2 diabetes. From the age of six years, II-3 and II-4 have been suffering from paroxysmal fatigue, salting craving, polydipsia and polyuria. They have no palpitation, constipation, or physical and mental retardation. II-5 (the third younger brother) has hyperuricemia and bilateral kidney stones, but he did not present any electrolytic disorder. III-1 (son) has suffered from renal dysfunction for three years and gout involving the first metatarsophalangeal joint of the left foot for one year, but he does not have kidney stones or any electrolytic disorders.

### 2.2. Genetic Analysis

**Figure 1:**
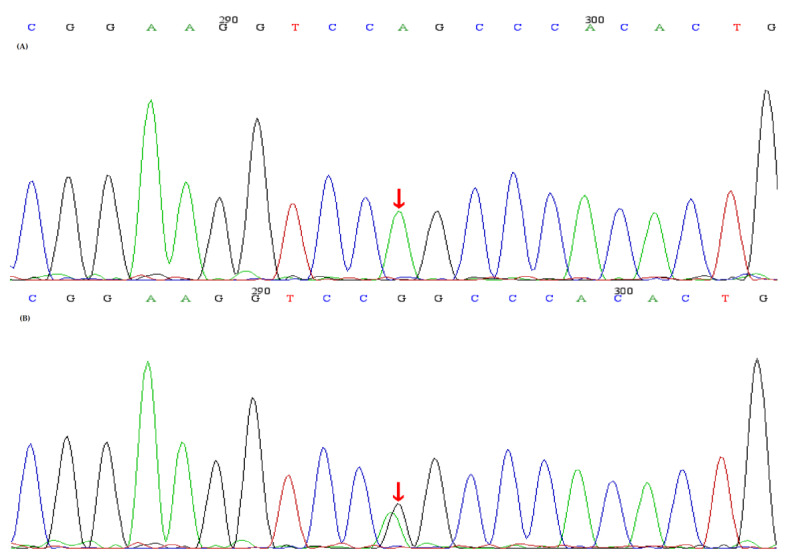
Sequence analyses of the *SLC12A3* gene and the identified *SLC12A3* mutations. (**A**) A homozygous mutation (red arrow) (c.248G>A, p.Arg83Gln)in *SLC12A3* was identified in the proband and II-2,II-4, (**B**) A heterozygous mutation (red arrow) (c.248G>A, p.Arg83Gln) in *SLC12A3* was identified in I-1,I-2,III-1.

**Figure 2:**
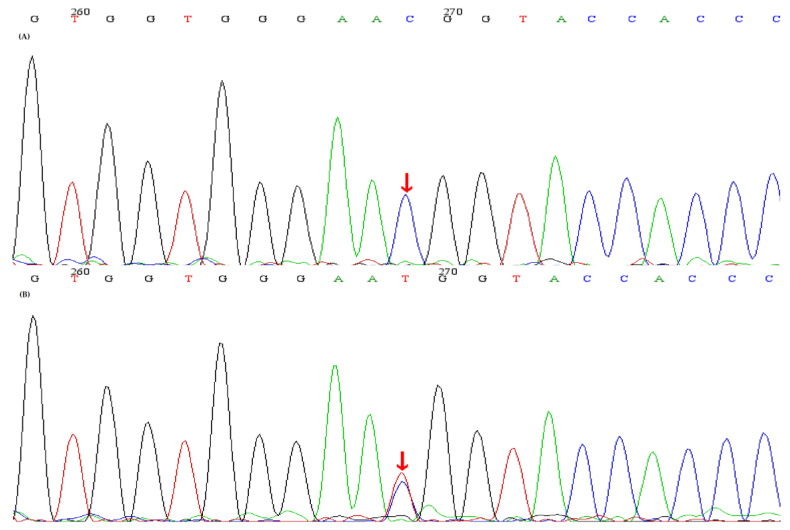
Sequence analyses of the *CLCNKB* gene and the identified *CLCNKB* mutations. (**A**) A homozygous mutation red arrow) (c.1171T>C, p.Trp391Arg) in *CLCNKB* was identified in the index patient and II-3, II-4. (**B**) A heterozygous mutation (red arrow) (c.1171T>C, p.Trp391Arg) in *CLCNKB* was identified in t I-1, I-2, II-5, III-1.

**Figure 3:**
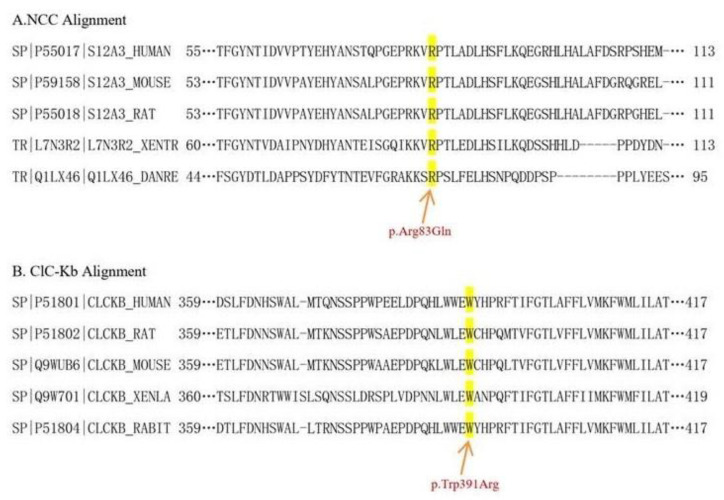
Phylogenetic conservation analysis. The amino acid residues altered by the corresponding variants studied here were highlighted. The phylogenetic conservation analysis revealed that the residues of 83 in NCC (**A**) and 391 in ClC-Kb (**B**) were both highly conserved across species.

**Figure 4:**
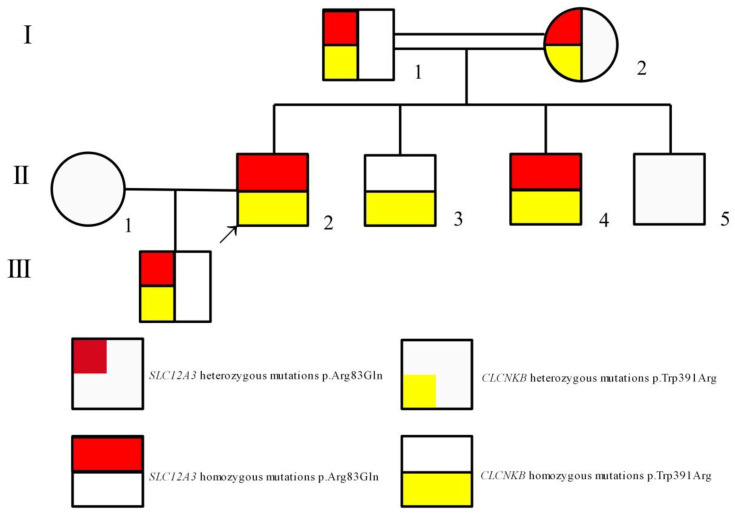
Pedigree of the family shows that the clinical phenotype cosegregates with simultaneous homozygous mutations in *SLC12A3* and *CLCNKB*. The arrow indicates the index patient (II-2). Males and females are indicated by squares and circles, respectively.

The study, in conformity with the Declaration of Helsinki, was approved by the ethics committee of the Second Affiliated Hospital, Zhejiang University School of Medicine. Written informed consents were obtained from all subjects. DNA was extracted from peripheral blood using a QIAamp DNA Blood Mini Kit (QIAGEN, Germany). The amplified DNA was captured with a Panel/Whole exome sequencing (WES) probe (MyGenostics, Beijing, China) following DNA Library Preparation. The panel applied in the proband (II-2) consisted of 62 known SLT related genes, including *SLC12A1, KCNJ1, CLCNKA, CLCNKB, BSND, CaSR, HNF1B* and *SLC12A3*. To identify the exonic deletions of *HNF1B*, capture copy-number variants (CapCNV) analysis was applied. WES, detecting all exon regions of over 20,000 genes, was applied in patient III:1. The enrichment libraries were sequenced on Illumina HiSeq X ten platform or paired-end read 150bp. After sequencing, bioinformatics analysis was used to select the potential pathogenic mutations. Variants were further annotated by ANNOVAR (http://annovar.openbioinformatics.org/en/latest/, (accessed on 13 January 2021)) and associated with multiple databases, including 1000 genome, ESP6500, dbSNP, EXAC, Inhouse (MyGenostics, Beijing, China), HGMD, and predicted by SIFT (http://sift.jcvi.org/, (accessed on 13 January 2021)), PolyPhen-2 (http://genetics.bwh.harvard.edu/pph2/, (accessed on 13 January 2021)), Mutation Taster (http://www.mutationtaster.org/, (accessed on 13 January 2021)), GERP++ (http://mendel.stanford.edu/SidowLab/downloads/gerp/index.html, (accessed on 13 January 2021)). Filtered candidate variants were confirmed by Sanger sequencing. The coding exons that contain the detected mutations were amplified with Ex Taq DNA polymerase (Takara, Dalian, China). Purified polymerase chain reaction (PCR) samples were sequenced on an ABI 3730 Genetic Analyzer (Applied Biosystems, Foster City, CA, USA). Sequence traces were analyzed using Mutation Surveyor (Softgenetics, PA, USA). The mutations of family members were confirmed by the same procedures. II-2 and II-4 harbored homozygous mutations (c.248G>A, p.Arg83Gln) in *SLC12A3*, I-1, I-2 and III-1 harbored heterozygous mutations (c.248G>A, p.Arg83Gln) in *SLC12A3* (Figure 1). II-2, II-3 and II-4 harbored homozygous mutations (c.1171T>C, p.Trp391Arg) in *CLCNKB*. I-:1, I-2, II-5 and III-1 harbored heterozygous mutations (c.1171T>C, p.Trp391Arg) in *CLCNKB* (Figure 2). II-2 and II-4 harbored dual homozygous mutations in *SLC12A3* and *CLCNKB*, and the diagnoses of GS and BS type III were, therefore, confirmed. II-3 harbors single homozygous mutations in *CLCNKB*, and the diagnosis of BS type III was thus identified. The variant of *SLC12A3* (p.Arg83Gln) was predicted to be probably-damaging by PolyPhen-2, disease-causing by Mutation Taster, and damaging by SIFT. This variant has been reported by Vargas-Poussou, et al [8] (Table 2). The residue of 83 in NCC was highly conserved across species (Figure 3).The variant of *CLCNKB*(p.Trp391Arg) was predicted to be probably-damaging by PolyPhen-2, disease-causing by Mutation Taster and damaging by SIFT (Table 2). This variant only occurred in the East Asian subpopulation, the allele frequency of the variant in the East Asian subpopulation was 0.0005514 [9]. The residue of 391 in ClC-Kb was highly conserved across species (Figure 3). Pedigree of the family showed the clinical phenotype cosegregates with homozygous *SLC12A3* and *CLCNKB* (Figure 4) mutations. However, no point mutations or intragenic exonic deletion of *HNF1B* were detected by CapCNV in all family members. WES failed to identify the causative gene mutation that was responsible for the early-onset gout and renal dysfunction of III-1.

### 2.3. Treatment and Follow Up

**Figure 5:**
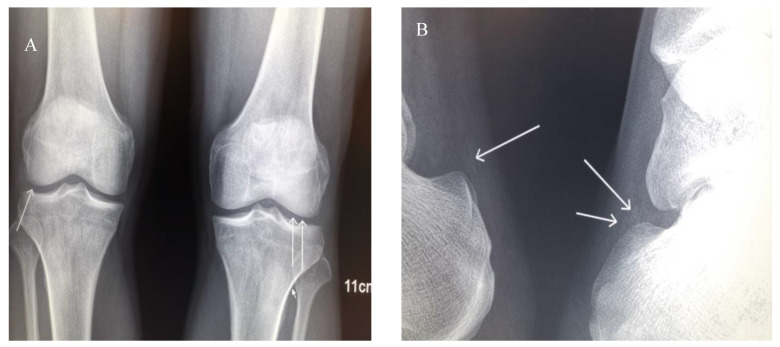
X-ray of joints. (**A**) Anteroposterior projections of the patient’s knees. Extensive chondrocalcinosis of the fibrocartilage of the medial and lateral menisci (arrows) is present. (**B**) Lateral projections of the ankles. Calcification of the Achilles tendons is present.

Potassium chloride (KCl) (2.0 g/day), spironolactone (40 mg/day) and potassium magnesium aspartate (8 tablets/day) were prescribed to II-2 to correct electrolytic disturbances. Benzbromarone (50 mg/day) was prescribed to correct hyperuricemia. On Sep 26th, 2019, serum chemistry analysis showed uric acid (UA) at 761 μmol/L, Creatinine (Cr) 145 μmol/L, K 2.9 mmol/L, and Mg 0.41 mmol/L. The arthralgia persisted. Febuxostat (50 mg/day) was, therefore, added to further lower UA. The arthritis in the PIP joint was completely resolved, and the arthralgia in bilateral knees and heels was slightly improved. X-rays of bilateral hands, shoulders, hips, knees and ankles revealed chondrocalcinosis (CC) in knees and Achilles tendons (Figure 5). The arthralgia in knees and heels was considered to be caused by CPPD resulting from hypomagnesemia. The dose of potassium magnesium aspartate was subsequently increased, and the remained arthralgia was gradually alleviated. On Apr 18th, 2020, the serum chemistry panel showed UA was 286 μmol/L, Cr 97 μmol/L, K 2.8 mmol/L, and Mg 0.63 mmol/L. KCl was prescribed to II-3 and II-4. Their fatigue subsequently improved, but they did not take KCl regularly. On Apr 18th, 2020, serum chemistry of II-3 showed UA 560 μmol/L, Cr 103 μmol/L, K 2.9 mmol/L, Mg 0.79 mmol/L. Serum chemistry of II-4 showed UA 580 μmol/L, Cr 150 μmol/L, K 1.8 mmol/L, Mg 0.79 mmol/L. Benzbromarone (50 mg/day) was administered to III-1 to correct hyperuricemia. On Apr 18th, 2020, serum chemistry of III-1 showed UA 397 μmol/L, Cr 105 μmol/L. The arthralgia was resolved.

## 3. Discussion

We described an exceptionally rare case in an inbred Chinese pedigree with simultaneous homozygous mutations in *SLC12A3* and *CLCNKB*. II-2 and II-4 with simultaneous homozygous mutations in *SLC12A3* and *CLCNKB* have phenotypes of mixed cBS and GS, and II-3 with one homozygous mutation in *CLCNKB* has phenotypes of cBS.

The ClC-Kb channel is expressed in the thick ascending limb (TAL), distal convoluted tubule (DCT) and collecting duct, where it transfers chloride (Cl^−^) ions to the basolateral side [2,10]. In some patients with BS type III, impaired ClC-Kb function in the TAL accounts for the Bartter phenotype, including hypercalciuria and isosthenuria, whereas impaired ClC-Kb function in the DCT accounts for the GLS phenotype, including hypocalciuria in other patients with BS type III [4]. The paracellular reabsorption of calcium in the TAL requires the electrochemical gradient created by NaCl transport in this tubule segment. Impaired ClC-Kb function in the TAL causes lower levels of Cl^−^ exit, NaCl reabsorption through the Na-K-2Cl cotransport (NKCC2) and subsequently calcium reabsorption [2,4]. In patients with GS, impaired NCC function in the DCT results in hypocalciuria. Diminished Na reabsorption across the luminal membrane of the DCT cell caused by impaired NCC function in DCT, coupled with continued efflux of intracellular Cl^−^ via basolateral Cl^−^ channels, results in the cell to hyperpolarize. This, in turn, stimulates entry of calcium into the cell via luminal voltage-activated Ca^2+^ channels. In addition, the lowering of intracellular Na+ concentration facilitates Ca^2+^ exit via a basolateral Na^+^/Ca^2+^ exchanger [11] Defective basolateral Cl^−^ exit in the DCT decreases NaCl reabsorption via the NCC, accounting for the GLS phenotype, including hypocalciuria in BS type III patients with impaired ClC-Kb function in the DCT [4].Accordingly, II-3 has isosthenuria and a significantly elevated uCa/Cr ratio, which both indicate the tubular location of the *CLCNKB* mutation was in the TAL instead of DCT. II-2 and II-4 have an additional *SLC12A3* mutation besides the *CLCNKB* mutation. DCT is distal to TAL, and thus the increased urinary calcium resulted from *CLCNKB* mutation in the TAL is then reabsorbed in the DCT due to the *SLC12A3* mutation. Finally, hypocalciuria is generated in II-2 and II-4. Accordingly, the GLS phenotypes of II-2 and II-4 are generated by the *SLC12A3* mutation rather than the *CLCNKB* mutation.

To the best of our knowledge, no cases with simultaneous homozygous mutations in the *SLC12A3* and *CLCNKB* genes have been previously reported. The first description of the simultaneous presence of *SLC12A3* and *CLCNKB* gene mutations was the case of two siblings (brother and sister) whose first symptoms appeared in infancy and childhood, respectively. Their serum magnesium and urine calcium excretion rate fluctuated, ranging from normal to low, indicating they had inconsistent hypomagnesemia and hypocalcinuria. Mutation analysis identified the simultaneous presence of heterozygous and compound heterozygous mutations in the *SLC12A3* and *CLCNKB* genes, respectively. They were diagnosed as cBS [12]. The mutation types of *SLC12A3* and *CLCNKB* were frameshift and missense, respectively. The brother developed proteinuria and had growth retardation due to growth hormone deficiency. The sister also had growth retardation. They both had normal renal function. Another female patient was reported to have growth retardation with persistent hypokalemia, hypomagnesemia, hypocalciuria, hypochloremic alkalosis and elevated levels of plasma renin and aldosterone. Her younger brother, father and paternal grandmother all had histories of mild-to-low levels of plasma potassium, which were rectified by potassium-rich foods. Gene sequencing revealed this patient carried a paternally inherited heterozygous mutation in *SLC12A*3 and maternally inherited heterozygous variants in both *CLCNKB* and *CLCNKA*. Based on clinical phenotypes, genetic evidence of the pedigree and previous reported studies, this case of GS indicates a digenetic inheritance of *SLC12A3* and *CLCNKB* that resulted in renal tubular dysfunction, perhaps due to a genetic double-hit mechanism. The putative pathogenicity of the *CLCNKB* p.L94I variant requires confirmation [13]. A 19-year-old male patient harbored a splice-site heterozygous mutation in *SLC12A3* and a missense heterozygous mutation in *CLCNKB*. His phenotype was consistent with severe GS [14]. In conclusion, a digenetic inheritance of heterozygous *SLC12A3* and *CLCNKB* mutations could cause SLT. I-1 also carried both heterozygous mutation in *SLC12A3* and *CLCNKB*, which may explain his hypokalemia. However, I-2 and III-1, both of whom carried the same mutations as I-1, had no electronic disorders.

Both GS and Type III BS have significant intrafamilial phenotype heterogeneity [3,4] Zelikovic reported a large Bedouin pedigree with a common homozygous missense mutation in the *CLCNKB* gene and the concomitant presence in the kindred of GS and cBS phenotypes. These findings demonstrated intrafamilial heterogeneity [11]. For the present pedigree, while both I-2 and II-4 carry the same dual homozygous mutations in *SLC12A3* and *CLCNKB*, II-2 had hypomagnesemia, gout and CPPD, whereas II-4 had normal magnesemia and kidney stones. Both of their phenotypes are consistent with mixed cBS and GS. It is hypothesized that variation in expression and/or function of any one of the channels or transporters participating in Cl^−^ transport in the TAL and the DCT may modify variable degrees of impairment in ClC-Kb function, thereby influencing the disease phenotype. Such a modifying effect could occur at the cellular/regulatory level, whereby one or more transport mechanisms are recruited to compensate for impaired ClC-Kb function or, alternatively, may be determined at the level of modifier gene [11].

BS type III has a high phenotypic variability with clinical presentations ranging from very severe salt-losing nephropathy with marked hypokalemia to almost asymptomatic presentation [15]. The type of mutation may influence the clinical presentation of BS type III. For the mutations in *CLCNKB*, missense mutations were more frequent in patients with less severe phenotypes. On the contrary, severe mutations (large deletions, frameshift, nonsense and essential splicing) were more frequent in patients with earlier onset and severe phenotypes [4]. II-3 carries a homozygous missense mutation in *CLCNKB* and has a mild phenotype of cBS. Therefore, his phenotype is consistent with his type of mutation in *CLCNKB*. II-2 and II-4 carry dual homozygous mutations in *SLC12A3* and *CLCNKB*. II-4 has renal dysfunction and very severe hypokalemia (serum K^+^ 1.8 mmol/L), which both indicate his phenotype is severe. II-2 has renal dysfunction, suffering from gout and CPPD. He has severe hypokalemia and moderate hypomagnesemia despite potassium and magnesium supplementation. Accordingly, his phenotype is also severe. The phenotypes of II-2 and II-4 are more severe than that of II-3 due to additional mutations in *SLC12A3*. GS is a milder disease compared with BS [4]. The type and position of mutation may influence the clinical presentation of GS. The mutations in *SLC12A3* that were associated with a severe presentation were, at least, the combination for one allele of a missense mutation that resulted in a nonfunctional intracellular retained protein or, even more frequent, a missplicing that resulted in a short transcript [16]. The missense mutation in *SLC12A3* that was identified here is located within the amino-terminal domain of NCC, which is not involved in defining affinity for ions and thiazides [17]. The central domain of NCC determines ion translocation and thiazide-binding specificity, which could provide a basis for the pathogenic role of these missense mutations [16,17]. Therefore, it is postulated that the mutation in *SLC12A3* that was identified here results in mild dysfunction of NCC. Taken together, the simultaneous mutations in *CLCNKB* and *SLC12A3* result in severe rather than very severe phenotypes, as patients take low dose of thiazide and loop diuretics rather than large dose of them.

In some patients with GS rather than BS, CPPD might develop due to long-term and profound hypomagnesemia [3]. CPPD may present with a variety of signs and symptoms, which may or may not be symptomatic. These include acute CPP crystal arthritis, an acute onset, and self-limiting synovitis with CPPD, previously known as “pseudogout” [5]. Therefore, arthritis resulting from CPPD is easily misdiagnosed as gout. Acute gouty arthritis is prevalent in patients with BS rather than GS [7]. As a result, it is difficult to identify the cause of arthralgia in patients with both BS and GS. II-2 had both hypomagnesemia and hyperuricemia. X-rays revealed the calcification of the Achilles tendons and knees, although the X-ray of hands was normal. UA lowering therapy only resolved arthralgia in the hands, but Mg supplementation resolved arthralgia in the ankles and knees. Hence, the arthralgia of the PIP joint of right index finger may have resulted from gout, whereas the arthralgia of ankles and knees resulted from CPPD. II-2 has both gout and CPPD, which indicates the phenotypes of mixed cBS and GS.

## 4. Conclusions

Simultaneous homozygous mutations in *SLC12A3* and *CLCNKB* gene are exceptionally rare. Consanguineous marriage should be strictly discouraged. GS and BS type III have significant intrafamilial phenotype heterogeneity. Both gout and CPPD should be considered and differentiated if arthralgia is observed in patients with GS and BS.

## Figures and Tables

**Table 1:** Characteristics of the pedigree.

Subject	I-1	I-2	II-2	II-3	II-4	II-5	III-1	Reference Range
Age (year)	69	69	48	46	44	42	25	
Sex	Male	Female	Male	Male	Male	Male	Male	
Serum biochemisty								
Cr (μmol/L)	115	77	99	92	129	93	117	62~115
UA^a^ (μmol/L)	605	288	998	433	324	458	582	150~420
Na (mmol/L)	142	140	138	140	133	139	140	137~147
Cl (mmol/L)	101	100	93	99	88	102	101	99~110
K (mmol/L)	3.4	3.9	2.6	3.0	2.4	4.1	4.2	3.5~5.3
Mg (mmol/L)	0.99	0.92	0.53	0.95	0.79	0.96	0.91	0.66~1.07
Ca (mmol/L)	2.26	2.38	2.15	2.13	2.33	2.19	2.33	2.0~2.50
eGFR ^b^ (mL/min/1.73 m^2^)	56.02	67.87	77.27	85.63	57.71	86.92	74.21	>90
Urine analysis								
Specific gravity	1.015	1.010	1.010	1.010	1.010	1.020	1.015	1.003~1.030
pH	5.5	5.50	7	6.5	7.5	5.5	5.5	4.5~8.0
Red blood cell (cells/μL)	111	0	4	1	1	8	3	0~15
Protein	0	0	0	0	0	0	0	Negative
24-h urine								
Urine volume (mL/24 h)	1450	750	3500	1600	1800	1700	1200	
K (mmol/24 h)	32.77	23.1	47.6	47.84	73.98	17.85	24.84	
Ca (mmol/24 h)			0.7	8.192	1.26			
Spot Ca/Cr ratio (mmol/mmol)	0.23	0.56	0.01	0.63	0.05	0.34	0.04	
Plasma Renin activity (upright) (ng/mL/h)			15.95	3.37	7.18			0.93~6.56
Plasma Aldosterone (upright) (pg/mL)			37.36	215.38	526.67			65.0~296.0
Arterial blood gas								
pH			7.49	7.41	7.47			7.35~7.45
PaO2 (mmHg)			74	105	104			80~100
PaCO2 (mmHg)			37	36.8	40.8			35~45
HCO3- (mmol/L)			27.9	22.8	29.6			21~28
BE ^c^ (mmol/L)			4.9	−1.1	5.9			+3~−3
Ultrasound of urinary tract	Left kidney stone	Normal	Normal	Normal	Bilateral kidney stones and right cyst	Bilateral kidney stones	Normal	

The labels in header of Table 1 follow the rules in drawing the pedigree tree. The ancient Roman numerals denote the generation of the pedigree and the Arabic numerals denote the subject in each generation. ^a^: UA: uric acid. ^b^: eGFR:estimated glomerular filtration rate, ^c^: BE: base excess.

**Table 2:** In silico prediction of the damaging effect of the two variants.

Gene	Exon	Transcript	Nucleotide Mutations	Amino Acid Variants	Variant Type	PolyPhen-2	Mutation Taster	SIFT	GERP
*SLC12A3*	1	NM_000085.5	c.248G>A	p.Arg83Gln	missense	1 (probably-damaging)	1 (disease-causing)	0 (damaging)	5.42 (conserved)
*CLCNKB*	12	NM_000339.3	c.1171T>C	p.Trp391Arg	missense	0.999 (probably-damaging)	1 (disease-causing)	0 (damaging)	4.59 (conserved)
SIFT: Sorting Intolerant From Tolerant, GERP: Gnomic Evolutionary Rate Profiling									

## Data Availability

Data sharing not applicable.
